# Data on evolutionary relationships of *Aeromonas hydrophila* and *Serratia proteamaculans* that attach to water tanks

**DOI:** 10.1016/j.dib.2017.10.073

**Published:** 2017-11-07

**Authors:** Ogueri Nwaiwu

**Affiliations:** a Division of Food Sciences, School of Biosciences, University of Nottingham, Sutton Bonington, Campus, LE12 5RD, College Road, Loughborough, Leicestershire, United Kingdom; b Alpha Altis, Ingenuity Center, University of Nottingham Innovation Park, Triumph Road, NG7 2TU, United Kingdom

**Keywords:** Bacteria attachment, Water tank, Biofouling, Evolution, Phylogeny

## Abstract

Here the data on evolutionary relationships of persistent bacteria from water tanks and their close relatives are shown. Curated sequences of the hypervariable region of ribosomal ribonucleic acid (rRNA) obtained from a strain of *Aeromonas hydrophila* and two strains of *Serratia proteamaculans* after searches in the GenBank® database were analyzed. The analysis which included 104 other bacteria strains, was carried out using molecular evolutionary genetic analysis (MEGA 7.0) software.

**Specifications Table**

**Table:** 

Subject area	*Microbiology*
More specific subject area	*Molecular phylogeny*
Type of data	*Figures*
How data was acquired	*Sequence search (16 Svedberg units or 16S) on GenBank*® *data base*
Data format	*Analyzed*
Experimental factors	*Statistical methods, bootstrap test*
Experimental features	*Evolutionary genetic analysis of curated sequences.*
Data source location	*Genbank*®.
Data accessibility	*Sequences used can be accessed in Genbank*® *using accession numbers*HG328350*-2. Sequences from 104 other isolates are available to the public via the accession numbers on the phylogenetic trees.*

**Value of the Data**

• Data shows phylogeny of water tank bacteria and other species from several sources.
• Selection of strains for comparative whole-genome analysis can be facilitated by the data.
• Data is useful for further investigations of weak or strong biofilm producers during fouling of water tanks.

## 1 Data

**Fig. 1:**
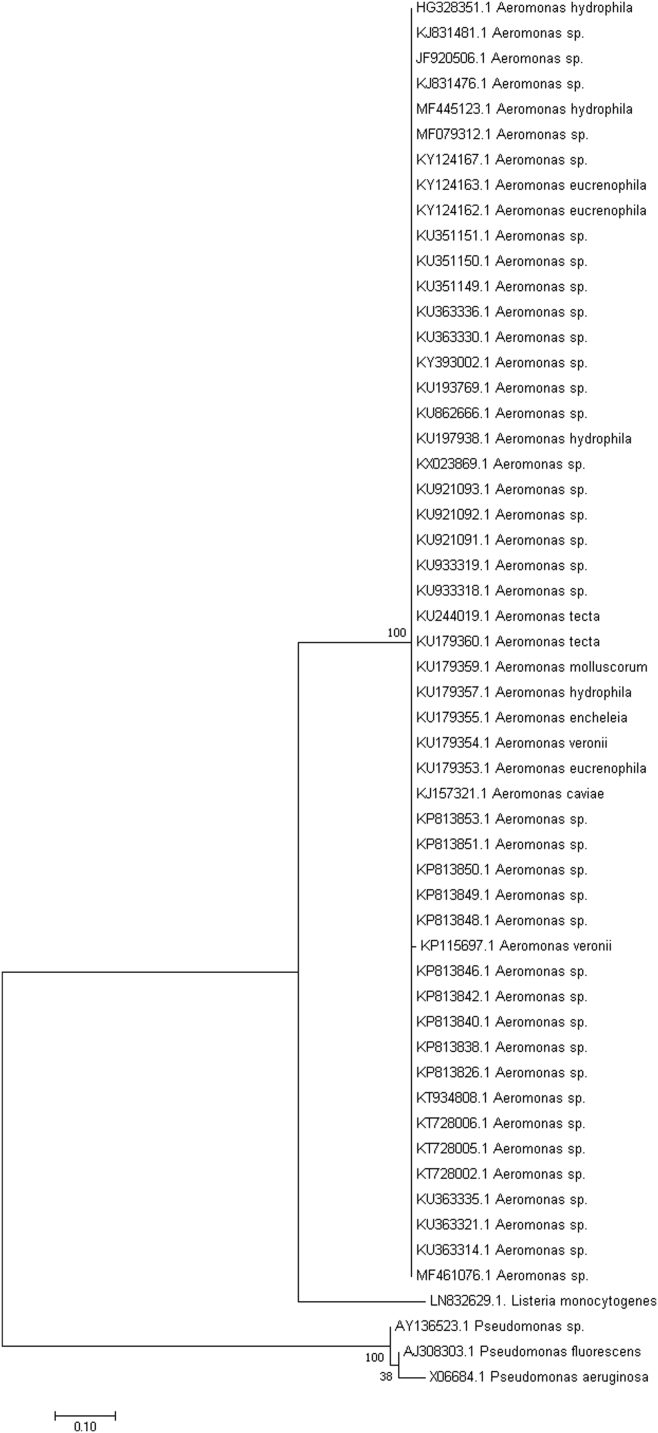
The evolutionary history of *A. hydrophila* (Accession No. HG328351) was determined by using the maximum likelihood statistical method to compare with 50 other sequences from close relatives in MEGA7 software. Species of *Listeria monocytogenes* and three species of *Pseudomonas* were also investigated. The tree with the highest log likelihood was selected and the percentages of trees in which the associated taxa clustered together in the bootstrap test (1000 replicates) are shown next to the branches. The tree was drawn to scale, with branch lengths measured in the number of substitutions per site. The analysis involved 55 nucleotide sequences. All positions containing gaps and missing data were eliminated.

**Fig. 2:**
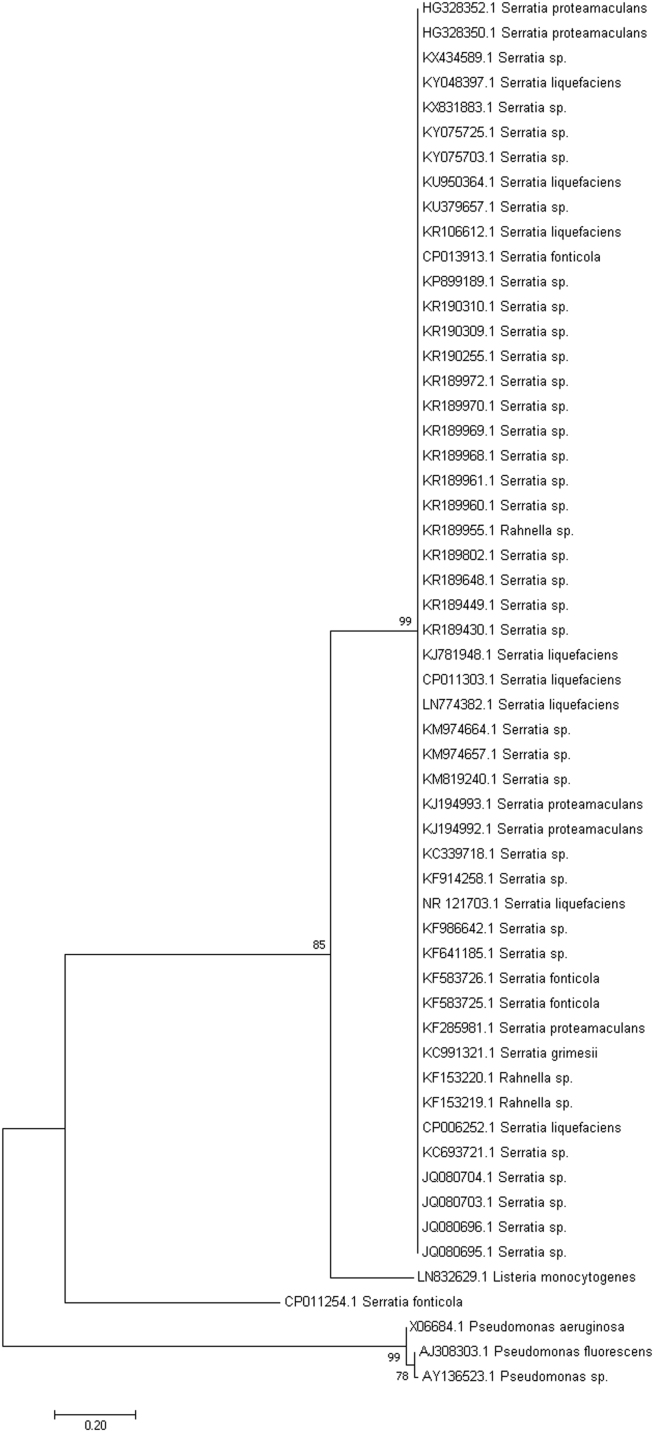
The evolutionary history of two strains of *Serratia proteamaculans* (HG328350 and HG3283502) was determined by using the maximum likelihood statistical method to compare with 50 other sequences from close relatives. The same outgroups and statistics shown in Fig. 1 were used. The analysis involved 56 nucleotide sequences.

The phylogeny data presented here have not been published in an initial study [1]. The 16S sequences from *A. hydrophila* (Fig. 1) and *S. proteamaculans* (Fig. 2) were compared with other isolates in order to gain more understanding of how they evolved. The clades formed after construction of phylogenetic trees show the evolutionary path of the sequences.

## 2 Experimental design, materials and methods

### 2.1 Molecular evolutionary genetics analysis (MEGA)

Sequences deposited in the Genbank® under accession numbers HG328351 (*A. hydrophila*), HG328350 and HG328352 (*S. proteamaculans*) from previous work [1] were analyzed.

Updated searches were carried out after which the top hits showing sequences from closely related culturable strains were selected for each genus and then subjected to phylogenetic analysis with MEGA software, version 7 [2]. After sequence alignment with ClustalW [3], the maximum likelihood statistical method based on the Tamura-Nei model [4] was used to generate phylogenetic trees. A total of 50 strains of closely related sequences from culturable strains were selected at random for each phylogenetic tree. Previously characterized 16S rRNA sequences that were used as outgroups included sequences from *Listeria monocytogenes* [5], *Pseudomonas aeruginosa* [6], *Pseudomonas fluorescens* [7] and a *Pseudomonas* species [8].
