# Supplementary material for: Data on evolutionary relationships of Aeromonas hydrophila and Serratia proteamaculans that attach to water tanks
Source: Data Brief. 2017 Nov 7;16:10–4. doi: 10.1016/j.dib.2017.10.073 (PMC5684427; doi:10.1016/j.dib.2017.10.073)
Supplement: Supplementary file 1 — Supplementary material [file mmc1.zip › Conflicts of interest.pdf]

Conflicts of interest:
none
